# Erratum: Distinct patterns of cognitive outcome in young children with autism spectrum disorder receiving the Early Start Denver Model

**DOI:** 10.3389/fpsyt.2023.1182124

**Published:** 2023-03-17

**Authors:** Frontiers Production Office

**Affiliations:** Frontiers Media SA, Lausanne, Switzerland

**Keywords:** autism spectrum disorders, early intervention, predictors, response to treatment, heterogeneity, minimal responder

An omission to the funding section of the original article was made in error. The following sentence has been added: “Open access funding was provided by the University of Geneva.”

The original article has been updated.

